# Joint frequency offset, time offset, and channel estimation for OFDM/OQAM systems

**DOI:** 10.1186/s13634-017-0526-4

**Published:** 2018-01-08

**Authors:** Ali Baghaki, Benoit Champagne

**Affiliations:** Department of Electrical and Computer Engineering, McGill University, 3480 University Street, Montreal, H3A 0E9 Canada

**Keywords:** OFDM/OQAM, Joint estimation, Filter bank multicarrier, Carrier frequency offset, Sampling time offset, Channel impulse response, Cramer-Rao bound

## Abstract

Among the multicarrier modulation techniques considered as an alternative to orthogonal frequency division multiplexing (OFDM) for future wireless networks, a derivative of OFDM based on offset quadrature amplitude modulation (OFDM/OQAM) has received considerable attention. In this paper, we propose an improved joint estimation method for carrier frequency offset, sampling time offset, and channel impulse response, needed for the practical application of OFDM/OQAM. The proposed joint ML estimator instruments a pilot-based maximum-likelihood (ML) estimation of the unknown parameters, as derived under the assumptions of Gaussian noise and independent input symbols. The ML estimator formulation relies on the splitting of each received pilot symbol into contributions from surrounding pilot symbols, non-pilot symbols and additive noise. Within the ML framework, the Cramer-Rao bound on the covariance matrix of unbiased estimators of the joint parameter vector under consideration is derived as a performance benchmark. The proposed method is compared with a highly cited previous work. The improvements in the results point to the superiority of the proposed method, which also performs close to the Cramer-Rao bound.

## Introduction

Due to the several benefits of multicarrier modulation (MCM) over single carrier modulation, the former has been considered as the primary choice in the physical layer implementation of telecommunication systems for quite a long time. Among the MCM family, orthogonal frequency division multiplexing (OFDM) has been largely studied and adopted in many wireless and wireline standards [1, 2]. Still, as an alternative and promising form of MCM for future generations of wireless networks, a variant of OFDM based on offset quadrature amplitude modulation (OFDM/OQAM) has attracted much research interest in recent years, due to its many advantages over classical OFDM, including higher spectral efficiency and reduced sensitivity to timing and frequency mismatch [3]. In spite of these advantages, accurate carrier frequency and timing synchronization along with channel estimation (for the purpose of equalization) remain of paramount importance for the successful application of OFDM/OQAM in practical systems.

There exist three main categories of synchronization and channel estimation methods for OFDM/OQAM systems: blind, semi-blind and pilot-based methods. While blind methods, e.g., [4–7], provide higher spectral efficiency by avoiding the overhead of training sequences, the requirement of a longer observation window for accurate estimation limits their tracking ability, rendering them less popular in most practical applications. In contrast, the semi-blind methods only require the transmission of a small number of parameters to resolve an estimation ambiguity, e.g., [8] and as such, they offer a useful trade-off between spectral efficiency and estimation accuracy. However, since in practical scenarios, the training symbol overhead needed to obtain a better estimation performance is usually tolerated, our focus here is on pilot-based synchronization and channel estimation.

Compared to channel estimation, pilot-based carrier frequency offset (CFO), and sampling time offset (STO) estimation has received less attention in the OFDM/OQAM literature. In [9], a maximum likelihood (ML) symbol timing estimator is derived by using two training symbols per burst transmission. In [10], using the same preamble structure, the authors extend this work by proposing a joint ML-based estimator of the CFO and STO. Then, to avoid the computational complexity of a two dimensional ML search, a feasible joint estimation method, called approximate maximum-likelihood (AML), is developed by assuming a small CFO and using only one OQAM preamble symbol per burst. The same authors, in [11], propose a joint least-squares (LS) CFO and STO estimation method by using two identical OFDM/OQAM pilot symbols per burst transmission. Therein, as a time-domain method, the estimation is performed before the analysis filter bank (AFB) at the receiver. In [12], by using a polyphase network implementation of OFDM/OQAM, the preloading technique, and a conjugate-symmetric preamble, the CFO and STO are separately estimated. The CFO estimation exploits the phase difference between the adjacent pilots while the frame detection and STO estimation are derived based on the conjugate-symmetry property. Moreover, in [13, 14], a joint CFO and STO estimation method is proposed by using a four-column preamble per burst transmission, which contains zeros in every other subcarrier and every other symbol time index.

In contrast to the CFO and STO estimation, during the past decade, many pilot-based channel estimation schemes have been proposed for OFDM/OQAM systems, which can be broadly classified into frequency domain and time domain methods. Frequency domain methods, e.g., [15–23], rely on the assumption that the symbol duration is much longer than the maximum channel delay spread. While these methods are generally characterized by a lower computational complexity, when the above condition is not satisfied, they will suffer from a performance degradation. Time domain methods, e.g., [24–26], attempt to estimate the channel impulse response (CIR) by using sequences of pilot tones. In [24], a time domain CIR estimator is proposed based on the multiple signal classification (MUSIC) and LS algorithms. In [25], a per-subchannel estimator is proposed in which the CIR on each subcarrier is estimated separately. In [26], the authors exploit pilot tone structures in OFDM/OQAM systems to derive two new CIR estimators, namely the linear minimum mean square error (LMMSE) and weighted least-square (WLS) estimators. The former exploits a priori knowledge of the CIR covariance matrix while the latter only requires the knowledge of the channel length; both methods are benchmarked against the Cramer-Rao bound (CRB). In a recent paper [27], based on a combination of the ideas of [15] and [28], a coded auxiliary pilot scheme is proposed for frequency domain channel estimation. The coded auxiliary pilots are carefully designed to compensate for the inherent imaginary interference of OFDM/OQAM.

Although the aforementioned synchronization and equalization problems have been separately addressed throughout the literature on OFDM/OQAM systems, only a few research papers can be found, e.g., [29], that are devoted to STO, CFO and channel estimation at the same time, let alone a joint estimation approach based on a unified criterion. In fact, to the best of our knowledge, a joint estimation method for OFDM/OQAM systems, accounting for all the three error sources, i.e., CFO, STO, and CIR, has not yet been developed. Hence, our focus in this paper is to develop and investigate a general estimation method to fill this need.

Specifically, a new formulation of the joint parameter estimation problem in OFDM/OQAM system is first introduced, which is based on splitting the interference term on the desired received pilot into adjacent pilot, non-pilot and noise contributions. Then, by assuming Gaussian noise and independent input symbols, a pilot-based joint ML estimator of CFO, STO, and CIR is derived. Such a general approach offers many advantages, including a unified framework for the estimation of multiple parameters using a common preamble/burst structure and the proper treatment of different types of interference in the estimator derivation. More importantly, a significant performance improvement is expected in the joint estimation of the aforementioned error sources as opposed to their separate estimation. Through numerical simulations of wireless OFDM/OQAM transmission over multipath fading channels, the proposed estimator is evaluated by comparing the accuracy of the resulting parameter estimates to that obtained with a selected benchmark approach among a few existing works where all the three error sources of our focus are estimated^1^, as well as to the CRB. The simulation results show that, the proposed method is capable of significant improvements in parameter estimation accuracy, performing close to the CRB. In turn, this improved performance leads to a lower bit error rate (BER) of the compensated (i.e., synchronized and equalized) transceiver system.

This paper is a more developed and improved version of our previous work [30] addressing the joint estimation problem under a more restrictive set of assumptions. Specifically, the new contributions of the current work include the following:

- In [30], orthogonality of the OFDM/OQAM analysis/synthesis filters in the complex domain is assumed, as opposed to orthogonality in the real domain only. The former condition leads to important simplifications in the derivation of the ML estimator, especially in the statistical properties of the subband noise and data interference.
  As a consequence of such simplifications, the resulting estimator in [30] only qualifies as an approximate ML estimator, although it shows performance improvements compared to earlier work. In contrast, herein, by invoking the true orthogonality condition of OFDM/OQAM in the real domain, we can strive for an exact ML-based estimator, which achieve even better estimation accuracy.
- Another important contribution of this paper is the analysis of the distributions of the subband noise and data interference terms in the general OFDM/OQAM framework, where the pilot tones can be scattered or appended as preamble to the data. In particular, we show that the subband noise contributions, after the real operation, are uncorrelated along the time and frequency axes, as a consequence of the exact orthogonality relation. Furthermore, we show through analysis and numerical simulations that the data interference terms are well modeled by a Gaussian distribution for which we derive the second order statistics. We further show that the data interference terms are only weakly correlated along the time and frequency axes. We conclude that with a carefully designed pilot distribution, their correlation can be confidently approximated as zero.
- Based on this reformulation of the problem and subsequent derivation of an accurate log-likelihood function for the received pilot tones, we derive in detail the CRB for the joint parameter estimation problem under consideration. The former plays a key role in demonstrating the near optimality of the newly derived joint ML-based estimator, whose performance (estimation error) comes within 1 dB or less from the bound.
- In addition to the above new theoretical contributions, the paper contains a number of improvements, including a computational complexity analysis and discussion of practical approaches to reduce implementation complexity.

The paper is organized as follows. Section 2 is dedicated to reviewing the OFDM/OQAM system model as implemented in this work. In Section 3, the joint ML estimator of the CFO, STO, and CIR is developed in details based on a new formulation. Several related aspects are also discussed, including: computational simplifications for efficient implementation; evaluation of computational complexity; CFO and STO compensation and channel equalization. The CRB on the unbiased estimator of the aforementioned parameters is derived in Section 4. The methodology used in our simulations and the results are provided in Section 5, while Section 6 concludes the paper. Appendices A and B provide important developments about statistical properties of the subband noise and data interference terms.

*Notations:* Bold-faced letters indicate vectors and matrices, e.g., ***A***. The (*i,j*)th entry of a matrix is represented by [***A***]_*i,j*_. The superscripts *T* and *H* stand for the transpose and Hermitian transpose of a vector or matrix, respectively. The operator ∗ represents a linear convolution while the superscript ^∗^ denotes complex conjugation. The identity and zero matrices are denoted by ***I*** and ***0***, respectively. The paraconjugate operation on a matrix function ***E***(*z*) is defined by $\boldsymbol {\tilde {E}}(z) = \boldsymbol {E}(1/z^{*})^{H}$. The operators E{.}, $\mathfrak {R}[.]$ and $\mathfrak {I}[.]$ stand for the expected value, real part and imaginary part of their arguments, respectively. The floor operation is denoted by ⌊.⌋ while ||.|| represents the second norm operation.

## Problem formulation

In this section, the OFDM/OQAM system model is presented along with its input-output relation over a frequency selective fading channel. The effects of the CFO and STO on the reconstructed signal are discussed and finally, the joint estimation problem for the CFO, STO, and CIR is stated.

### OFDM/OQAM System Model

**Fig. 1:**
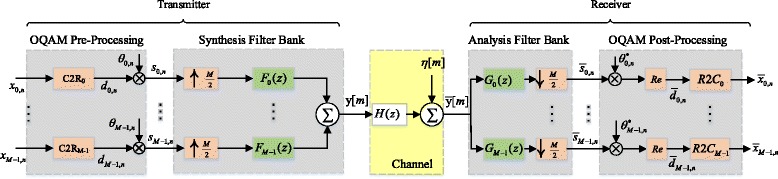
OFDM/OQAM system model

The OFDM/OQAM system model, as implemented in this work and commonly used in the literature, e.g., [31], is illustrated in Fig. 1. OFDM/OQAM makes use of a specific filter bank structure where the upsampling and downsampling factor equals half the number of subcarriers, denoted by *M*. At each input symbol time, with symbol duration *T*_*s*_, a vector of discrete input symbols is loaded on the *M* available subcarriers. The latter are separated in frequency by *F*_*s*_=1/*T*_*s*_, so that the system occupies a total bandwidth of *W*=*MF*_*s*_.

On the transmitter side, let $x_{k,n}\in \mathcal {A}$ denote the complex valued symbols at the input, where *k*∈{0,1,…,*M*−1} is the frequency index, $n\in \mathbb {Z}$ is the symbol time index, and $\mathcal {A}$ is the digital constellation from which the symbols are drawn. In the first stage of pre-processing, each *x*_*k,n*_ is converted to a pair of real symbols, *d*_*k,n*_, according to the following equations,

1 $$ {}\begin{array}{ccc} & \thinmuskip=0mu \medmuskip=0mu d_{k,2n}= \left\{\begin{array}{cc} \mathfrak{R}\left[x_{k,n}\right], & k \ \text{even} \\ \mathfrak{I}\left[x_{k,n}\right], & k \ \text{odd} \end{array}\right. & d_{k,2n+1}= \left\{\begin{array}{cc} \mathfrak{I}\left[x_{k,n}\right], & k \ \text{even} \\ \mathfrak{R}\left[x_{k,n}\right], & k \ \text{odd} \end{array}.\right. \end{array}  $$

This complex-to-real (C2R) operation doubles the sampling rate of the subcarrier signals. The second stage of pre-processing involves multiplication of the real OQAM symbols, *d*_*k,n*_, by the sequence $\theta _{k,n}=e^{j \frac {\pi }{2}(k+n)}$, which results in complex symbols

2 $$ s_{k,n}=d_{k,n} \theta_{k,n}=d_{k,n}e^{j \frac{\pi}{2}(k+n)}.  $$

It is notable that the OQAM symbol duration is *T*_*s*_/2, i.e., one half of the input symbol duration. Next, in the synthesis filter bank (SFB), the input subcarrier signals *s*_*k,n*_ are first upsampled by *M*/2, and then passed through synthesis filters with finite impulse responses (FIR) *f*_*k*_[*m*] of length *L*_*p*_ and corresponding system functions $F_{k}(z)=\sum _{m=0}^{L_{p}-1}f_{k}[m]z^{-m}$. Finally, the individual filter outputs are added together to form the baseband output *y*[*m*] as follows,

3 $$ y[m]= \sum_{k=0}^{M-1} \sum_{n\in \mathbb{Z}}s_{k,n} \;f_{k}\left[m-\frac{nM}{2}\right].  $$

In a practical implementation of OFDM/OQAM, the output signal *y*[*m*] is passed through a pulse shaping filter and up-converted to an appropriate frequency band for transmission over the physical medium. In this work, however, we consider an equivalent baseband channel model for simplicity. Specifically, the channel is modeled as a linear time-invariant system with FIR *h*[*l*] of length *Q*, and corresponding system function $H(z)=\sum _{l=0}^{Q-1} h[l] z^{-l}$. The filter length *Q* is proportional to the channel delay spread *τ*_*ds*_, that is, *Q*=⌊*Mτ*_*ds*_/*T*_*s*_⌋+1. The channel coefficients *h*[*l*] are assumed to remain constant during the transmission time of one data block of *N* symbols, i.e., block duration plus overall processing delay of the transceiver system. Finally, the channel output is corrupted by additive white Gaussian noise (AWGN) *η*[*m*], assumed to be circularly complex with zero mean and variance $E[|\eta [m]|^{2}]=\sigma _{\eta }^{2}$. A more detailed discussion of the effects of fading channels, CFO and STO is provided in Subsection 2.2.

On the receiver side, let $\bar {y}[m]$ denote the received baseband signal after transmission through the noisy channel. In the analysis filter bank (AFB), signal $\bar {y}[m]$ is passed through the analysis filters with FIR *g*_*k*_[*m*] of length *L*_*p*_ and corresponding system functions^2^$G_{k}(z)=\sum _{m=-L_{p}+1}^{0} g_{k}[m] z^{-m}$, whose outputs are downsampled by *M*/2 afterwards. The resulting symbols at the output of the AFB can be represented as

4 $$ \bar{s}_{k,n} =\sum_{m=-\infty}^{\infty}\bar{y}[m]g_{k}\left[\frac{nM}{2}-m\right],  $$

where the range of summation over *m* is determined by the finite support of the subband FIR filters.

The symbols $\bar {s}_{k,n}$ then pass through the first post-processing stage, which involves multiplication by the sequence $\theta _{k,n}^{*}$ followed by taking the real part, i.e.,

5 $$ \bar{d}_{k,n}=\mathfrak{R}\left[\bar{s}_{k,n}\; \theta_{k,n}^{*}\right]=\mathfrak{R}\left[\bar{s}_{k,n} \; e^{-j \frac{\pi}{2} (k+n)}\right].  $$

The second post-processing stage is the real-to-complex (R2C) conversion, where two consecutive real valued symbols are combined into a complex one as follows,

6 $$ \bar{x}_{k,n}= \left\{\begin{array}{cc} \bar{d}_{k,2n}+j\bar{d}_{k,2n+1}, & k \ \text{even}, \\ \bar{d}_{k,2n+1}+j\bar{d}_{k,2n}, & k \ \text{odd}. \end{array}\right.  $$

We consider a complex-valued, uniform modulated filter bank, where the subchannel filters are all generated from a common low-pass prototype filter, *p*[*m*], by means of exponential modulation as follows,

7 $$\begin{array}{*{20}l} f_{k}[m]=p[m]e^{j\frac{2\pi km}{M}}, \:\: g_{k}[m]=f^{*}_{k}[-m], \end{array} $$

where *k*∈{0,1,…,*M*−1}.

The prototype filter used in this work is a near perfect reconstruction (NPR), real-valued linear phase (symmetric) FIR low-pass filter with length *L*_*p*_ and support region *m*∈{0,1,…,*L*_*p*_−1}. It is derived by using the frequency sampling technique, as in [31], with overlap factor *K*, so that its non-zero coefficients can be represented in closed form as

8 $$\begin{array}{*{20}l} p[m]&=\frac{\alpha}{KM}\left(1+2\sum\limits_{l=1}^{K-1}(-1)^{l} A[l] \cos\left(\frac{2\pi l}{KM}(m+1)\right)\right), \end{array} $$

where the coefficients *A*[*l*] satisfy *A*[*l*]^2^+*A*[*K*−*l*]^2^=1 for *l*=1,2,…,⌊*K*/2⌋ and *α* is a normalization factor such that $\sum _{m} p[m]^{2} = 1$.

In particular, for the adopted value of the overlap factor, i.e., *K*=4, we have

9 $$ A[1]=x,\; A[2]=1/\sqrt{2}, \; A[3]=\sqrt{1-x^{2}},  $$

where *A*[1]=*x* can be determined by using various optimization criteria. Since the LS criterion is used in this work (i.e., minimizing stopband energy), we set *A*[1]=0.97741677 according to Table 1 in [31]. Since the prototype filter is linear-phase (symmetric), the overall processing delay of the complete OFDM/OQAM transceiver system will be *L*_*p*_*T*_*s*_/*M*.

By using the paraconjugates of the synthesis filters as the analysis filters in the receiver, as specified in (7), the orthogonality condition of the transceiver system can be expressed as

10 $$\begin{array}{*{20}l} \mathfrak{R} \left\{\theta_{k,n}^{*}\theta_{k',n'} \sum\limits_{m=-\infty}^{\infty}f_{k}^{*}\left[\!m-\frac{n M}{2}\!\right] \: f_{k'}\left[\!m-\frac{n' M}{2}\!\right]\!\right\} \approx \delta_{kk'} \delta_{nn'} \;, \end{array} $$

where $\phantom {\dot {i}\!}\delta _{kk^{\prime }}$ denotes the Kronecker delta function [3, 32, 33]^3^.

### Effects of fading channel, carrier frequency offset, and sampling time offset

In addition to channel fading and additive noise, as illustrated in Fig. 1, the received signal $\bar {y}[m]$ at the front-end of the receiver will be affected by CFO due to oscillator mismatch or Doppler effect, as well as STO due to imperfect sampling. These effects can be mathematically modeled as

11 $$\begin{array}{*{20}l} \bar{y}[m]& = \left(h[m]*y[m-\tau_{0} ]\right)e^{-j 2 \pi \mu_{0} m / M} + \eta[m]\;\;\;\;\;\;  \end{array} $$

12 $$\begin{array}{*{20}l} & = \left(\sum\limits_{l=0}^{Q-1}{h[l]y[m-l-\tau_{0}]}\right) e^{-j 2 \pi \mu_{0} m / M} +\eta[m], \end{array} $$

where *τ*_0_ is the normalized STO^4^ with respect to the sampling period at the baseband transmitter output, *T*_*s*_/*M*, and *μ*_0_ is the normalized CFO with respect to *F*_*s*_, the subcarrier frequency spacing. It is worth mentioning that, similar to previous works on this subject (e.g., [12, 13]) the second-order effects, i.e., those of CFO, STO, and CIR on one another, have been neglected in this model. It has been observed through simulations that these effects are, indeed, negligible.

**Fig. 2:**
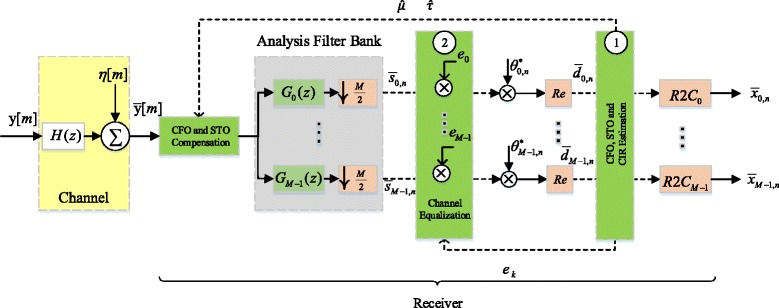
Block diagram of the OFDM/OQAM receiver with ML-based compensation

From (12), (4), and (5), useful expressions can be obtained for the real-valued output symbols $\bar {d}_{k,n}$ that appear in the OQAM post-processing module on the receiver side in Fig. 2,^5^. Specifically,

13 $$  \bar{d}_{k,n} = \zeta_{k,n} + \eta_{k,n},  $$

where *ζ*_*k,n*_ represents the contribution from the transmitted data (pilot and information symbols) as given by

14 $$\begin{array}{*{20}l} \zeta_{k,n}= & \mathfrak{R} \left\{\theta_{k,n}^{*} \left(\sum\limits_{m=-\infty}^{\infty} (h[m] *y[m-\tau_{0}])\right.\right.\\ &\left.\left.{\vphantom{\sum_{m=-\infty}^{\infty}}}e^{-j 2 \pi \mu_{0} m / M}g_{k}\left[\frac{nM}{2}-m \right]\right) \right\}, \end{array} $$

and *η*_*k,n*_ represents the additive noise (i.e., the contribution from *η*[*m*]) passed through analysis filter bank and first post-processing stage, as given by

15 $$ \eta_{k,n}= \mathfrak{R} \left\{\theta_{k,n}^{*} \left(\sum\limits_{m=-\infty}^{\infty}\eta[m]\; g_{k}\left[\frac{nM}{2}-m\right]\right) \right\}.  $$

Substituting (3) into (14) and using (7), *ζ*_*k,n*_ can be further developed as follows,

16 $$  \zeta_{k,n}= \mathfrak{R} \left\{\sum\limits_{l=0}^{Q-1} h[l] \lambda_{k,n}(l,\mu_{0},\tau_{0}) \right\},  $$

where we define

17 $$  \lambda_{k,n}(l,\mu_{0},\tau_{0})=\sum\limits_{k'=0}^{M-1} \sum\limits_{n'=-\infty}^{\infty} d_{k',n'} \gamma_{k,n}^{k',n'}(l,\mu_{0},\tau_{0}),  $$

18 $$\begin{array}{*{20}l}  \gamma^{k',n'}_{k,n}(l,\mu_{0},\tau_{0}) =&\ \theta^{*}_{k,n}\theta_{k',n'} \sum\limits_{m=-\infty}^{\infty}f_{k'}\left[m-l-\tau_{0}- \frac{n'M}{2}\right]\\ &f_{k}^{*}\left[m- \frac{nM}{2}\right] e^{-j2 \pi \mu_{0} m/ M}. \end{array} $$

The term $\gamma ^{k',n'}_{k,n}(l,\mu _{0},\tau _{0})$ in (18), known as ambiguity function [34], characterizes the level of the ‘intrinsic interference’ of the *n*^′^th real input sample from the *k*^′^th subband, $\phantom {\dot {i}\!}d_{k^{\prime },n^{\prime }}$, on the *n*th output sample from the *k*th subband, through the *l*th channel tap, *h*[*l*], in the presence of CFO, *μ*_0_, and STO, *τ*_0_. In the special case *l*=*μ*_0_=*τ*_0_=0, the quantity $\gamma _{k,n}^{k',n'}(0,0,0)$ describes the level of complex orthogonality of the analysis/synthesis filters of the OFDM/OQAM transceiver system up to a multiplicative factor $\theta _{k',n'} \theta ^{*}_{k,n}$. The values of $\gamma _{k,n}^{k',n'}(0,0,0)$ for the filter bank adopted in this work, with the prototype filter *p*[*m*] and its parameters as described in Section 2.1, are given in Table 1. We note that due to the finite length of the subband filters *f*_*k*_[*m*], the summation in (18) is, in fact, performed over a finite range.

**Table 1:** Transmultiplexer response $\gamma _{k,n}^{k',n'}(0,0,0)$ of the OFDM/OQAM system using Bellanger’s (also known as PHYDYAS) filter [41, 42] with *K*=4 (numerical values truncated to 4 digits)

	*n*^′^=*n*−4	*n*^′^=*n*−3	*n*^′^=*n*−2	*n*^′^=*n*−1	*n*^′^=*n*	*n*^′^=*n*+1	*n*^′^=*n*+2	*n*^′^=*n*+3	*n*^′^=*n*+4
*k*^′^=*k*−1	0.0107 j	0.0506 j	0.1246 j	0.1980 j	0.2283 j	0.1980 j	0.1246 j	0.0506 j	0.0107 j
*k*^′^=*k*	-0.0002	0.0765 j	-0.0005	0.5720 j	1	-0.5720 j	-0.0005	-0.0765 j	-0.0002
*k*^′^=*k*+1	-0.0107 j	0.0506 j	-0.1246 j	0.1980 j	-0.2283 j	0.1980 j	-0.1246 j	0.0506 j	-0.0107 j

For the same reason, the range of summation over the symbol time index *n*^′^ in (17) is also finite.

### Problem statement

If estimates of the CFO and STO are available, they can be compensated at the receiver front-end to avoid their degrading effects. Likewise, if estimates of the CIR coefficients are available, they can be used on the receiver side to design a set of subband equalizers to compensate for the distortion caused by the multipath fading channel^6^. The estimation and compensation of these imperfections is critical to achieve the low level of bit error rate (BER) required for the practical operation of multi-carrier modulation in broadband communication systems.

The main focus of this work, therefore, lies in the joint estimation and compensation of above imperfections for the OFDM/OQAM system. To this end, the use of pilot-based estimation is preferred over the blind approach, since the latter generally requires a longer data record to achieve a desired level of accuracy, which in turns increases the computational complexity and limits applications to static or very slowly time-varying channels. Furthermore, the framework of point estimation theory is employed here, where the parameters under estimation are modeled as unknown, yet deterministic quantities, i.e., no prior distribution is assumed. By transmitting a sequence of known pilot tones, and observing the received sequence over a given time interval, our specific interest lies in developing and investigating the properties of the joint ML estimator of the CFO *μ*_0_, STO *τ*_0_ and CIR *h*[*l*] for the generic OFDM/OQAM transceiver system illustrated in Fig. 1 and described in mathematical terms in Section 2.1. We shall denote the resulting ML estimates by $\hat {\mu }$, $\hat {\tau }$ and $\hat {h}[l]$, respectively.

## Joint estimation

In this section, we first introduce our proposed approach to the estimation problem by splitting a received pilot symbol into pilot, data, and noise contributions. Next, we formulate and develop a pilot-based joint ML estimator of the CFO, STO and CIR. We then present possible simplifications to reduce the implementation complexity of the resulting joint ML estimator and discuss its computational requirements. Finally, we explain how the jointly estimated parameters can be used to compensate the detrimental effects of STO, CTO, and CIR.

### Structure of received pilots

In this work, for convenience in analysis, a real OQAM symbol at time *n* is defined as the ordered set of *M* subband symbols *d*_*k,n*_ for *k*∈{0,1,…,*M*−1}, as they appear at the output of the C2R modules in the pre-processing stage of the SFB in Fig. 1 To allow for flexibility in the application of the derived pilot-based estimation method, we consider a general framework for the allocation of pilots. Specifically, within a burst of *N* consecutive symbols (e.g., from time *n*=0 to *N*−1), a total of *N*_*p*_ symbols, denoted as $d_{k,n} \equiv d_{k,n}^{\;p}$, are transmitted as pilots with time and frequency indexes $(k,n) \in \mathcal {P}$, where $\mathcal {P} \subseteq \{0,\ldots,M-1\}\times \{0,\dots,N-1\}$. In order to simplify the mathematical developments, we may consider a rectangular distribution of pilots, as in $\mathcal {P}=\mathcal {S} \times \mathcal {T}$ where $\mathcal {S} \subseteq \{0,\ldots,M-1\}$ and $\mathcal {T} \subseteq \{0,\ldots,N-1\}$; however, the extension of the resulting estimator to an arbitrary time-frequency grid $\mathcal {P}$ is straightforward. By definition, the pilot symbols $d_{k,n}^{\;p}$ are deterministic quantities, known to the receiver side.

For clarity in the presentation, the data or information symbols (i.e., non-pilot) are denoted as $d_{k,n} \equiv d_{k,n}^{\;d}$ where $(k,n) \notin \mathcal {P}$. These symbols, unknown to the receiver, are modeled as independent and identically distributed (i.i.d.) random variables with zero-mean and variance $\frac {1}{2}\sigma _{x}^{2}$.

As mentioned earlier, a demodulated pilot symbol on the receiver side of the OFDM/OQAM system, assuming a general pilot distribution $\mathcal {P}$ which is scattered among the data symbols, consists of additive contributions from surrounding pilot symbols, surrounding data symbols and noise. Specifically, for the case $(k,n) \in \mathcal {P}$, the real-valued output symbol $\bar {d}_{k,n} \equiv \bar {d}_{k,n}^{\;p}$ in (13) can be written as

19 $$\begin{array}{*{20}l} \bar{d}^{\;p}_{k,n}= \;\zeta_{k,n}^{\;p}+ \zeta_{k,n}^{\;d} + \eta_{k,n}&, \end{array} $$

where $\zeta ^{\;p}_{k,n}$ and $\zeta _{k,n}^{\;d}$, respectively, denote the contributions from surrounding pilots and data. The pilot contribution can be expressed as

20 $$\begin{array}{*{20}l} \zeta_{k,n}^{\;p}&= \; \mathfrak{R}\left\{ \sum\limits_{l=0}^{Q-1} h[l] \bar{\lambda}_{k,n}(l,\mu_{0},\tau_{0})\right\}, \end{array} $$

where

21 $$\begin{array}{*{20}l} \bar{\lambda}_{k,n}(l,\mu_{0},\tau_{0})&=\sum\limits_{(k',n') \in \mathcal{P}} d^{\;p}_{k',n'} \gamma_{k,n}^{k',n'}(l,\mu_{0},\tau_{0}). \end{array} $$

The data contribution, which can be interpreted as “data interference”, can be expressed as

22 $$\begin{array}{*{20}l} \zeta_{k,n}^{\;d}&=\sum\limits_{(k',n') \notin \mathcal{P}} d_{k',n'}^{\;d} \mathfrak{R} \left\{ \sum\limits_{l=0}^{Q-1} h[l] \gamma_{k,n}^{k',n'}(l,\mu_{0},\tau_{0}) \right\}. \end{array} $$

In the sequel, we use the described splitting of a received symbol into pilot, data and noise contributions to develop the joint ML estimator of CFO, STO and CIR for a general pilot-data distribution. When the received symbol $\bar {d}_{k,n} = \bar {d}_{k,n}^{\;p}$ corresponds to a transmitted pilot, in the aforementioned formulation in (20) and (21), the various terms $d_{k',n'}^{\;p}$ represent the contribution from the corresponding transmitted pilot $\left (\mathrm {~i.e.~} d_{k,n}^{\;p}\right)$, as well as, depending on the pilot distribution, possible contributions from surrounding pilot symbols $\left (\mathrm {~i.e.~}, d_{k',n'}^{\;p} \mathrm {~for~} (k',n') \in \mathcal {P} \mathrm {~and~} (k',n') \neq (k,n)\right)$. Since the pilots are known symbols, this part can be accounted for as a deterministic component (albeit dependent on the unknown parameters *μ*_0_, *τ*_0_ and *h*[*l*]) in the derivation of the ML estimator. To further proceed with this derivation, we therefore need to characterize the statistical properties of the noise term *η*_*k,n*_ and the data contribution term $\zeta _{k,n}^{\;d}$ in the decomposition (19) of the received pilot symbol $\bar {d}_{k,n}^{\;p}$.

From the AWGN assumption made earlier on the additive channel noise *η*[*m*], and the linearity of the processing operations involved in the OFDM/OQAM receiver, it follows that the subband noise contribution *η*_*k,n*_ is a jointly Gaussian (real) random process in the variables (*k,n*). Furthermore, on account of the orthogonality property of the analysis and synthesis filters, as stated in (10), it follows that the various random variables *η*_*k,n*_ (for different pairs (*k,n*)) are uncorrelated and therefore statistically independent. Specifically, it can be shown (see Appendix A) that

23 $$\begin{array}{*{20}l} \mathrm{E} \: \{\eta_{n,k}\eta_{n',k'}\}= \frac{\sigma^{2}_{\eta}}{2} \delta_{kk'}\delta_{nn'}. \end{array} $$

The data contribution term $\zeta _{k,n}^{\;d}$ in (22) is unknown to the receiver and therefore should be modeled as a (real-valued) random process. Based on the assumptions made above on $d_{k,n}^{\;d}$, we note that the expression (22) involves the weighted sum of a large number of statistically independent, zero-mean terms $d_{k',n'}^{\;d}$. Hence, invoking the central limit theorem, we shall assume that $\zeta _{k,n}^{d}$ in (22) can be conveniently modeled as zero-mean (real-valued) Gaussian random process. This assumption is further investigated in Appendix B. In addition, under mild assumptions usually satisfied in applications, it can be shown that the random variables $\zeta _{k,n}^{\;d}$ (for different pairs (*k,n*)) are nearly uncorrelated. Specifically (see Appendix B), we have that

24 $$\begin{array}{*{20}l} \mathrm{E}\:\left\{\zeta^{\:d}_{k,n} \zeta^{\:d}_{k',n'}\right\}&\approx\: \frac{\sigma_{\zeta^{d}}^{2}}{2}\; \delta_{k {k'}}\delta_{n {n'}}, \end{array} $$

25 $$\begin{array}{*{20}l} \sigma_{\zeta^{d}}^{2}&\,=\,\frac{\sigma_{x}^{2}}{2}\!\sum\limits_{(k',n') \notin \mathcal{P}}\!\left(\!\mathfrak{R}\left\{\!\sum\limits_{l} h[l]\gamma^{k',n'}_{k,n}(l,\mu_{0},\tau_{0})\right\}\!\right)^{2}. \end{array} $$

From (19), (23), and (24) by introducing $v_{k,n} = \zeta _{k,n}^{\;d} + \eta _{k,n}$ and assuming that the data and the additive noise are statistically independent, it follows that the terms *v*_*k,n*_ are independent Gaussian random variables with variance $\sigma ^{2}_{v}=\sigma ^{2}_{\zeta ^{d}}+\frac {\sigma ^{2}_{\eta }}{2}$. Finally, by substituting *v*_*k,n*_ in (19) we will have

26 $$ \bar{d}^{\;p}_{k,n}=\zeta_{k,n}^{\; p}+v_{k,n},  $$

which provides a convenient basis for the derivation of the coveted ML estimator.

### Pilot-based joint ML estimator

Focusing on the pilot contribution in (20), we have

27 $$\begin{array}{*{20}l} \zeta_{k,n}^{\;p}&=\mathfrak{R}\left\{\sum\limits_{l=0}^{Q-1} h[l] \bar{\lambda}_{k,n}(l,\mu_{0},\tau_{0})\right\} \end{array} $$

28 $$\begin{array}{*{20}l} &= \sum\limits_{l=0}^{Q-1}\left(h^{R}[l] \bar{\lambda}^{R}_{k,n}(l,\mu_{0},\tau_{0}) - h^{I}[l] \bar{\lambda}_{k,n}^{I}(l,\mu_{0},\tau_{0})\right), \end{array} $$

where the superscripts *R* and *I* are used in the sequel to identify the real and imaginary parts of the underlying quantity. Hence, by letting

$$\begin{aligned} \boldsymbol{\lambda}^{R}_{k,n}(\mu_{0},\tau_{0}) & = \left[\bar{\lambda}^{R}_{k,n}(0,\mu_{0},\tau_{0}),\bar{\lambda}^{R}_{k,n}(1,\mu_{0},\tau_{0}), \ldots, \bar{\lambda}^{R}_{k,n}(Q-1,\mu_{0},\tau_{0})\right], \\ \boldsymbol{\lambda}^{I}_{k,n}(\mu_{0},\tau_{0}) & = \left[ \bar{\lambda}^{I}_{k,n}(0,\mu_{0},\tau_{0}),\bar{\lambda}^{I}_{k,n}(1,\mu_{0},\tau_{0}), \ldots, \bar{\lambda}^{I}_{k,n}(Q-1,\mu_{0},\tau_{0})\right], \\ \mathbf{h}^{R} &=[h^{R}[0],h^{R}[1],\ldots,h^{R}[Q-1]]^{T}, \\ \mathbf{h}^{I} &=[h^{I}[0],h^{I}[1],\ldots,h^{I}[Q-1]]^{T}, \end{aligned} $$

we can obtain the relationship between the received symbol and the channel taps as

29 $$\begin{array}{*{20}l} \zeta_{k,n}^{\;p}= \;& \Big[\boldsymbol{{\lambda}}^{R}_{k,n}(\mu_{0},\tau_{0}) \: \: \: \boldsymbol{{\lambda}}^{I}_{k,n}(\mu_{0},\tau_{0})\Big]_{1 \times 2Q} \; {\left[ \begin{array}{c} \mathbf{\:\:\:h}^{R}\\ \mathbf{-h}^{I} \end{array} \right]}_{2Q \times 1}. \end{array} $$

By stacking $\bar {d}^{\;p}_{k,n}$, $\boldsymbol {\lambda }^{R}_{k,n}(\mu _{0},\tau _{0})$ and $\boldsymbol {\lambda }^{I}_{k,n}(\mu _{0},\tau _{0})$, and *v*_*k,n*_ over the time index *n* and then over the frequency index *k*, we arrive at the following matrix-vector equation,

$$\begin{array}{*{20}l}  \left[\boldsymbol{\bar{D}^{p}}\right]_{N_{p} \times 1}=&{ \underbrace{\left[\boldsymbol{\Lambda}^{R}(\mu_{0},\tau_{0}) \:\: \boldsymbol{\Lambda}^{I}(\mu_{0},\tau_{0})\right]}_{\boldsymbol{\Lambda'}(\mu_{0},\tau_{0})}}_{N_{p} \times 2Q} \: {\underbrace{\left[ \begin{array}{c} \mathbf{\:\:\:h}^{R} \\ \mathbf{-h}^{I} \end{array}\right]}_{\mathbf{h'}}}_{2Q \times 1}\\ &+\left[\boldsymbol{V}\right]_{N_{p} \times 1}. \end{array} $$

As a result of the AWGN assumption and the ensuing assumptions on the noise and data interference terms *η*_*k,n*_ and $\zeta ^{\;d}_{k,n}$, ***V*** will be a real Gaussian random vector with zero mean and a nearly diagonal covariance matrix $\boldsymbol {C}_{\boldsymbol {V}}=\mathrm {E}[\boldsymbol {V}\boldsymbol {V}^{T}]\approx \sigma ^{2}_{v}\boldsymbol {I}$. Similarly, for given values of *μ*_0_, *τ*_0_ and **h**, the observation $\boldsymbol {\bar {D}^{p}}$ is also a Gaussian random vector with mean ***Λ***^***′***^(*μ*_0_,*τ*_0_)*h*^′^ and covariance $\boldsymbol {C}_{\boldsymbol {\bar {D}^{p}}}\approx \sigma ^{2}_{v}\boldsymbol {I}$. Hence, the probability density function (PDF) of $\boldsymbol {\bar {D}^{p}}$ can be presented as,

30 $$ \begin{aligned} f(\boldsymbol{\bar{D}^{p}};\mu_{0},\tau_{0},\mathbf{h'})=& \frac{1}{\sqrt{(2\pi)^{N_{p}}\det(\boldsymbol{C}_{\boldsymbol{\bar{D}^{p}}})}} \\ &\exp\!\left[\,-\,\frac{1}{2}\!\left(\boldsymbol{\!\bar{D}^{p}}\,-\, \boldsymbol{\Lambda'}(\mu_{0},\tau_{0})\mathbf{h'}\right)^{T}\!\!\boldsymbol{C}_{\boldsymbol{\bar{D}^{p}}}^{-1}\left(\boldsymbol{\bar{D}^{p}}\!\,-\, \boldsymbol{\Lambda'}\!(\mu_{0},\tau_{0})\mathbf{h'}\right)\!\right]. \end{aligned}  $$

Thus, the log-likelihood function (LLF) is written, up to a constant, as,

31 $$ \begin{aligned} \mathcal{L}\left(\boldsymbol{\bar{D}^{p}};\mu_{0},\tau_{0},\mathbf{h'}\right) &= \log\left(f\left(\boldsymbol{\bar{D}^{p}};\mu_{0},\tau_{0},\mathbf{h'}\right)\right)\\ &= -\frac{1}{2\sigma^{2}_{v}}\left[\boldsymbol{\bar{D}^{p}}- \boldsymbol{\Lambda'}(\mu_{0},\tau_{0})\mathbf{h'}\right]^{T}\left[\boldsymbol{\bar{D}^{p}}-\boldsymbol{\Lambda'}(\mu_{0},\tau_{0})\mathbf{h'}\right]. \end{aligned}  $$

The joint ML estimators of the CFO, CIR and STO can be obtained by maximizing the derived LLF with respect to the parameters *μ*_0_, *τ*_0_ and *h*^′^. Let the unknown search parameters for CFO and STO be denoted by *μ* and *τ*. By fixing *μ* and *τ* and varying *h*^′^ in $\mathbb {C}^{2Q}$, the LLF achieves its maximum at

32 $$  \tilde{\mathbf{h'}}(\mu,\tau)=\boldsymbol{\Lambda'}(\mu,\tau)^{\dag}\boldsymbol{\bar{D}^{p}},  $$

where ***Λ***^***′***^(*μ*,*τ*)^*†*^=(***Λ***^***′***^(*μ*,*τ*)^*H*^***Λ***^***′***^(*μ*,*τ*))^−1^***Λ***^***′***^(*μ*,*τ*)^*H*^ is the pseudo-inverse of ***Λ***^***′***^(*μ*,*τ*). By substituting the resulting channel guess of (32) into the LLF we can obtain the CFO and STO estimates using a two-dimensional search according to

33 $$ (\hat{\mu},\hat{\tau})=\underset{(\mu,\tau)}{\arg\max}\, \mathcal{L}(\boldsymbol{\bar{D}^{p}};\mu,\tau,\tilde{\mathbf{h'}}).  $$

The ML estimate of the CIR can be obtained by substituting the estimates $(\hat {\mu },\hat {\tau })$, resulting in

34 $$ \hat{\mathbf{h'}}=\tilde{\mathbf{h'}}(\hat{\mu},\hat{\tau})=\boldsymbol{\Lambda'}(\hat{\mu},\hat{\tau})^{\dag}\boldsymbol{\bar{D}^{p}}.  $$

### Computational simplifications

Herein, three simplifications are introduced in computing $\bar {\lambda }_{k,n}(l,\mu _{0},\tau _{0})$ in (21) to speed up the calculation of the LLF (31) significantly. To this end, we first consider the term $\gamma ^{k',n'}_{k,n}(l,\mu _{0},\tau _{0})$ in (18), whose definition includes a summation over the length (pretty large) of the prototype filter *p*[*m*]. Since for the filter banks, we have *f*_*k*_[*m*]=*p*[*m*]*e*^*j*2*πkm*/*M*^, we can write

35 $$\begin{array}{*{20}l} \gamma^{k',n'}_{k,n}(l,\mu_{0},\tau_{0}) =&\ \theta^{*}_{k,n}\theta_{k',n'}\varphi^{n,n'}_{k-k'}(l,\mu_{0},\tau_{0}) \\&\exp\! \left\{\!-\frac{j 2 \pi}{M}\! \left(\!k'(l+\tau_{0}) \,+\,\frac{M}{2}(n'k'-nk)\right)\!\right\}\!, \end{array} $$

where letting *α*=*k*−*k*^′^

36 $$\begin{array}{*{20}l}  \varphi^{n,n'}_{\alpha}(l,\mu_{0},\tau_{0})=& \sum\limits_{m=-\infty}^{\infty}p\left[m-l-\tau_{0}-\frac{n'M}{2}\right]\\ &p\left[m-\frac{nM}{2}\right] e^{\frac{-j2 \pi}{M} m (\mu_{0} +\alpha)}. \end{array} $$

In this way, instead of calculating $\gamma ^{k',n'}_{k,n}(l,\mu _{0},\tau _{0})$ for all the possible pairs of (*k*^′^,*k*), it is sufficient to compute $\varphi ^{n,n'}_{\alpha }(l,\mu _{0},\tau _{0})$ for only possible values of *k*−*k*^′^=*α* and find the corresponding $\gamma ^{k',n'}_{k,n}(l,\mu _{0},\tau _{0})$ by multiplication with a discrete phase factor as in (35). The number of possible values of *α* depends on the distribution of the pilots over the frequency axis.

In the second simplification, regarding the calculation of the interference from the surrounding pilots, $\bar {\lambda }_{k,n}(l,\mu _{0},\tau _{0})$, we might assume that, owing to the excellent spectral containment of the prototype filters, the main source of the CFO-induced interference on each subband is due to its first few neighboring subbands; i.e, the interference from more distant subbands is negligible. Hence, to derive the total interference from subbands *k*^′^ on the subband with index *k* in (21), it suffices to take into the account the interference from a few neighboring pilot-carrying subbands on each side of the *k*th subband. Consequently, (21) can be approximated as

37 $$\begin{array}{*{20}l} \bar{\lambda}_{k,n}(l,\mu_{0},\tau_{0})\approx \sum\limits^{k+\beta}_{\substack{{k'=k-\beta}\\ {(k',n') \in (\mathcal{P}) }}} d_{k',n'}^{\: p} \gamma^{k',n'}_{k,n}(l,\mu_{0},\tau_{0}), \end{array} $$

where in practice, the value of *β* can be set^7^to 2.

To reduce the complexity of the estimator even further and make the two-dimensional search for CFO-STO more practical, the third compromise is to only consider the first few channel taps in computing pilot contribution in (27). This is due to the fact that these taps contribute the most to the entire power of the channel. By considering the implemented channel as described in Section 5.1, this reduces the running time of the proposed method approximately by three times. As our experiments confirm, this simplification only introduces a marginal degradation to the performance of the estimator, while maintaining it still significantly superior to that of the benchmark.

It is notable that, from (32) to (34), a second iteration of this estimation method can be performed by running a two-dimensional search over (*μ*,*τ*) using the obtained channel estimate and continuing to obtain a new set of estimates. However, our experiments indicate that the improvement gained by performing a second iteration is not significant enough to justify the additional complexity. Indeed, as it will be seen from the results, performing close to the CRB, a single iteration suffices to provide a significant improvement over the benchmark method.

As the LLF in (31) provides a closed form solution to the estimation problem, the multi-dimensional ML estimation is reduced to a two-dimensional search over the unknown CFO and STO, *μ* and *τ*. The search is performed in two stages, namely, a coarse search followed by a fine one in proximity of the coarse estimate. The decisive factor in the complexity of the proposed ML estimator is the number of operations required for each evaluation of the LLF. This is approximately calculated as $ C\simeq 8 M Q N_{p}^{2}+8Q^{2} N_{p}+\mathcal {O}(QN_{p})$ complex-valued operations where the first term is the cost of forming **Λ**^′^(*μ*,*τ*), the second term is the cost of QR decomposition to solve $\bar {\boldsymbol {D}}^{p}= \mathbf {\Lambda }'(\mu,\tau)\tilde {\mathbf {h}}'(\mu,\tau) $ and the third term is the cost of forming the LLF. Since the first term is dominant for typical *N*_*p*_ and *Q*, we conclude that the overall complexity is proportional to the squared number of pilot tones in the burst. This complexity evaluation is based on the case where none of the aforementioned practical simplifications above in Subsection (3.3) are applied. Employing these simplifications reduces the complexity of the practical implementation by a factor of $\mathcal {O}\left (QM^{2}\right)$, where the first two foregoing simplifications each reduce the complexity by a factor $\mathcal {O}(M)$ and the third one by a factor $\mathcal {O}(Q)$.

### Compensation of the estimated parameters

Figure 2 illustrates the block diagram of the OFDM/OQAM receiver as implemented in the proposed method for estimation and compensation of CFO, STO and CIR. First, after passing through the AFB and multiplication by $\theta ^{*}_{k,n}$ and real taking, the received symbols are used to obtain the ML estimates of the CFO, STO and CIR. The CFO and STO estimates are then fed back to correct the signal at the front-end of the receiver. The CFO-STO compensated signal can be written as

38 $$\begin{array}{*{20}l} \bar{y}^{c}[m]= W_{I}[m;\hat{\tau}] * \left(\bar{y}[m]e^{\frac{j2 \pi \hat{\mu} m}{M}} \right), \end{array} $$

where $W_{I}[m;\hat {\tau }]$ represents the hamming-windowed sinc fractional-delay interpolation filter used for STO simulation [35]. Next, the CFO-STO corrected symbols pass through the AFB again where, this time, a single-tap per subcarrier equalization is performed based on the DFT of the estimated CIR according to the following equation,

39 $$ e_{k}=\frac{1}{\hat{H}(z)}\bigg|_{z=e^{j 2 \pi k/M}},  $$

where *e*_*k*_ for *k*∈{0,…,*M*−1} is the coefficient of the equalizer for subband *k* and $ \hat {H}(z)=\sum _{l=0}^{Q-1}\hat {h}[l]z^{-l}$, $\hat {h}[l]$ being the estimated CIR coefficients. It is notable that although a single-tap per subcarrier equalizer is used here, generalizations to other, more advanced types of equalizers are possible. This simple equalization scheme inverts the channel at the center frequency of the corresponding subcarrier and it works well in mildly selective channels as long as the number of subcarriers is sufficiently large [36]. The equalized symbols can be represented as,

40 $$ \bar{s}^{c}_{k,n}=\left(\sum\limits_{m=-\infty}^{\infty}\bar{y}^{c}[m]g_{k}\left[\frac{nM}{2}-m\right]\right)e_{k}.  $$

The final received symbols are then obtained by undergoing the OQAM post-processing stage.

It is notable that the formulation of the problem through the linear equations obtained from the LLF of the received pilots, leads to performing the channel estimation in the time domain. Furthermore, as mentioned earlier in Section 1, the channel estimation methods in time domain do not make assumption on the subchannels being almost flat. In contrast, the channel equalization has been performed in the frequency domain by using one-tap-per-subcarrier scheme, which is a common equalization technique in MCM systems including OFDM and OFDM/OQAM. In addition to simplicity, this technique allows for flexibility of equalization in a multi-user scenario where different subchannels need to be equalized separately.

## Joint Cramer-Rao bound analysis based on the Gaussian assumption for data interference $\zeta ^{\;d}_{k,n}$

In this section, assuming known transmitted symbols, i.e., pilots, the CRB on the covariance matrix of unbiased estimators of the CFO, STO and CIR are derived [37]^8^. Letting ***θ*** be the vector including the unknown (real) parameters we have,

41 $$ \boldsymbol{\theta}=\left[\mu,\tau,(\mathbf{h}^{R})^{T},\left(\mathbf{h}^{I}\right)^{T}\right]^{T}.  $$

Therefore, ***θ*** holds (2*Q*+2) real entries with indexes denoted by *a* and *b*∈{1,2,…,2*Q*+2}. The Fisher information matrix (FIM), $\mathcal {J}$, is then (2*Q*+2)×(2*Q*+2). For the covariance matrix ***C***_***V***_, since for all *a* in the aforementioned interval *∂****C***_***V***_/*∂θ*_*a*_=**0**, the entries of FIM are given by,

42 $$\begin{array}{*{20}l} [\mathcal{J}(\boldsymbol{\theta})]_{a,b} =&-\mathrm{E} {\left\lbrace \frac{\partial^{2} \mathcal{L}(\boldsymbol{{\bar{D}^{p}}};{\boldsymbol{\theta}})} {\partial \theta_{a} \partial \theta_{b}}\right\rbrace} \\=& \frac{\partial (\boldsymbol{\Lambda}'(\mu,\tau)\mathbf{h}')^{T}} {\partial \theta_{a}}\boldsymbol{C}^{-1}_{\bar{D}^{p}} \frac{\partial (\boldsymbol{\Lambda}'(\mu,\tau)\mathbf{h}')}{\partial \theta_{b}}, \end{array} $$

which results in,

43 $$\begin{array}{*{20}l} [\mathcal{J}(\boldsymbol{\theta})]_{a,b}= \frac{1}{\sigma^{2}_{v}} \left[\sum\limits_{(k,n)\in \mathcal{P}} \frac{\partial \left(\boldsymbol{\lambda}^{R}_{k,n}(\mu,\tau)\mathbf{h}^{R}-\boldsymbol{\lambda}^{I}_{k,n}(\mu,\tau)\mathbf{h}^{I}\right)}{\partial \theta_{a}} \right.\\ \left.\frac{\partial \left(\boldsymbol{\lambda}^{R}_{k,n}(\mu,\tau)\mathbf{h}^{R}-\boldsymbol{\lambda}^{I}_{k,n}(\mu,\tau)\mathbf{h}^{I}\right)}{\partial \theta_{b}}\right]. \end{array} $$

Letting $A \triangleq \boldsymbol {\lambda }^{R}_{k,n}(\mu,\tau)\mathbf {h}^{R}-\boldsymbol {\lambda }^{I}_{k,n}(\mu,\tau)\mathbf {h}^{I} $, the partial derivative of *A* with respect to *θ*_*a*_ for four different ranges of the indexes *a* and *b*, namely *a*=1, *a*=2, 3≤*a*≤*Q*+2 and *Q*+3≤*a*≤2*Q*+2 can be written as,

44 $$\begin{array}{*{20}l} \frac{\partial A}{\partial \theta_{1}}=\frac{\partial A}{\partial \mu}=\frac{\partial \boldsymbol{\lambda}^{R}_{k,n}(\mu,\tau)}{\partial \mu} \mathbf{h}^{R} -\frac{\partial \boldsymbol{\lambda}^{I}_{k,n}(\mu,\tau)}{\partial \mu} \mathbf{h}^{I},  \end{array} $$

45 $$\begin{array}{*{20}l} \frac{\partial A}{\partial \theta_{2}}=\frac{\partial A}{\partial \tau}=\frac{\partial \boldsymbol{\lambda}^{R}_{k,n}(\mu,\tau)}{\partial \tau} \mathbf{h}^{R} -\frac{\partial \boldsymbol{\lambda}^{I}_{k,n}(\mu,\tau)}{\partial \tau} \mathbf{h}^{I}. \end{array} $$

Also, for 3≤*a*≤*Q*+2,

46 $$\begin{array}{*{20}l} \frac{\partial A}{\partial \theta_{a}}=\frac{\partial A}{\partial {\mathrm{h}}^{R}[l]}=\boldsymbol{\lambda}^{R}_{k,n}(\mu,\tau)\frac{\partial \mathbf{h}^{R}}{\partial {\mathrm{h}}^{R}[l]} = \bar{\lambda}^{R}_{k,n}(l,\mu,\tau), \end{array} $$

where *l*=*a*−2. In addition, for *Q*+2≤*a*≤2*Q*+2

47 $$\begin{array}{*{20}l} \frac{\partial A}{\partial \theta_{a}}\!=\frac{\partial A}{\partial {\mathrm{h}}^{I}[l]}=-\boldsymbol{\lambda}^{I}_{k,n}(\mu,\tau)\frac{\partial \mathbf{h}^{I}}{\partial {\mathrm{h}}^{I}[l]} =- \bar{\lambda}^{I}_{k,n}(l,\mu,\tau). \end{array} $$

Thus, $\mathcal {J}(\boldsymbol {\theta })$ can be written as

48 $$\begin{array}{*{20}l} \mathcal{J}(\boldsymbol{\theta})=\left[ \begin{array}{cccc} J_{1,1} & J_{1,2} & \boldsymbol{\epsilon} & \kappa \\ J_{2,1} & J_{2,2} & \boldsymbol{\phi} & \boldsymbol{\rho} \\ (\boldsymbol{\epsilon})^{T} & (\boldsymbol{\phi})^{T} & \boldsymbol{\chi} & \boldsymbol{\psi} \\ (\boldsymbol{\kappa})^{T} & (\boldsymbol{\rho})^{T} & (\boldsymbol{\psi})^{T} & \boldsymbol{\zeta} \end{array} \right], \end{array} $$

where according to (42)–(47) we have,

49 $$\begin{array}{*{20}l} J_{1,1}=\frac{1}{\sigma^{2}_{v}} \left[\sum\limits_{(k,n)\in \mathcal{P}} \left| \frac{\partial (\boldsymbol{\lambda}^{R}_{k,n}(\mu,\tau))}{\partial \mu}\mathbf{h}^{R} - \frac{\partial (\boldsymbol{\lambda}^{I}_{k,n}(\mu,\tau))}{\partial \mu}\mathbf{h}^{I} \right|^{2} \right], \end{array} $$

50 $$\begin{array}{*{20}l} J_{2,2}=\frac{1}{\sigma^{2}_{v}} \left[\sum\limits_{(k,n)\in \mathcal{P}} \left| \frac{\partial (\boldsymbol{\lambda}^{R}_{k,n}(\mu,\tau))}{\partial \tau}\mathbf{h}^{R} - \frac{\partial (\boldsymbol{\lambda}^{I}_{k,n}(\mu,\tau))}{\partial \tau}\mathbf{h}^{I} \right|^{2} \right], \end{array} $$

51 $$\begin{array}{*{20}l} J_{1,2}=\frac{1}{\sigma^{2}_{v}}& \left[\!\sum\limits_{(k,n)\in \mathcal{P}} \left(\frac{\partial \left(\boldsymbol{\lambda}^{R}_{k,n}(\mu,\tau)\right)}{\partial \mu}\mathbf{h}^{R} - \frac{\partial \left(\boldsymbol{\lambda}^{I}_{k,n}(\mu,\tau)\right)}{\partial \mu}\mathbf{h}^{I}\right)  \right.\\ & \left.\left(\frac{\partial \left(\boldsymbol{\lambda}^{R}_{k,n}(\mu,\tau)\right)}{\partial \tau}\mathbf{h}^{R} - \frac{\partial \left(\boldsymbol{\lambda}^{I}_{k,n}(\mu,\tau)\right)}{\partial \tau}\mathbf{h}^{I} \right) \right], \end{array} $$

52 $$\begin{array}{*{20}l} J_{2,1}=J_{1,2}. \end{array} $$

Also, ***ε*** is a 1×*Q* vector whose entries can be written as

53 $$\begin{array}{*{20}l} \boldsymbol{\epsilon}_{1,b}=&\frac{1}{\sigma^{2}_{v}}\sum\limits_{(k,n)\in \mathcal{P}} \left(\frac{\partial \left(\boldsymbol{\lambda}^{R}_{k,n}(\mu,\tau)\right)}{\partial \mu} \mathbf{h}^{R} - \frac{\partial \left(\boldsymbol{\lambda}^{I}_{k,n}(\mu,\tau)\right)}{\partial \mu} \mathbf{h}^{I} \right)\\ &\bar{\lambda}^{R}_{k,n}(b,\mu,\tau), \end{array} $$

54 $$\begin{array}{*{20}l} \boldsymbol{\kappa}_{1,b}=&-\frac{1}{\sigma^{2}_{v}}\sum\limits_{(k,n)\in \mathcal{P}} \left(\frac{\partial (\boldsymbol{\lambda}^{R}_{k,n}(\mu,\tau))}{\partial \mu} \mathbf{h}^{R} - \frac{\partial (\boldsymbol{\lambda}^{I}_{k,n}(\mu,\tau))}{\partial \mu} \mathbf{h}^{I} \right)\\ &\bar{\lambda}^{I}_{k,n}(b,\mu,\tau). \end{array} $$

Moreover, ***ϕ*** is a 1×*Q* vector whose entries can be represented as

55 $$\begin{array}{*{20}l} \boldsymbol{\phi}_{1,b}=&\frac{1}{\sigma^{2}_{v}}\sum\limits_{(k,n)\in \mathcal{P}} \left(\frac{\partial (\boldsymbol{\lambda}^{R}_{k,n}(\mu,\tau))}{\partial \tau} \mathbf{h}^{R} - \frac{\partial (\boldsymbol{\lambda}^{I}_{k,n}(\mu,\tau))}{\partial \tau} \mathbf{h}^{I} \right)\\ &\bar{\lambda}^{R}_{k,n}(b,\mu,\tau), \end{array} $$

56 $$\begin{array}{*{20}l} \boldsymbol{\rho}_{1,b}=&-\frac{1}{\sigma^{2}_{v}}\sum\limits_{(k,n)\in \mathcal{P}} \left(\frac{\partial (\boldsymbol{\lambda}^{R}_{k,n}(\mu,\tau))}{\partial \tau} \mathbf{h}^{R} - \frac{\partial (\boldsymbol{\lambda}^{I}_{k,n}(\mu,\tau))}{\partial \tau} \mathbf{h}^{I} \right)\\ &\bar{\lambda}^{I}_{k,n}(b,\mu,\tau). \end{array} $$

In addition, ***χ***, ***ψ*** and ***ζ*** are *Q*×*Q* matrices with the following entries,

57 $$\begin{array}{*{20}l} \boldsymbol{\chi}_{a,b}=\frac{1}{\sigma^{2}_{v}}\sum\limits_{(k,n)\in \mathcal{P}} \bar{\lambda}^{R}_{k,n}(a,\mu,\tau)\bar{\lambda}^{R}_{k,n}(b,\mu,\tau), \end{array} $$

58 $$\begin{array}{*{20}l} \boldsymbol{\psi}_{a,b}=-\frac{1}{\sigma^{2}_{v}}\sum\limits_{(k,n)\in \mathcal{P}} \bar{\lambda}^{R}_{k,n}(a,\mu,\tau) \bar{\lambda}^{I}_{k,n}(b,\mu,\tau), \end{array} $$

59 $$\begin{array}{*{20}l} \boldsymbol{\zeta}_{a,b}=\frac{1}{\sigma^{2}_{v}}\sum\limits_{(k,n)\in \mathcal{P}} \bar{\lambda}^{I}_{k,n}(a,\mu,\tau)\bar{\lambda}^{I}_{k,n}(b,\mu,\tau). \end{array} $$

The CRB of an unbiased estimator of ***θ***, denoted as $\boldsymbol {\hat {\theta }}$, is expressed as Cov$(\hat {\boldsymbol {\theta }}) \geq \mathcal {J}(\boldsymbol {\theta })^{-1}$. As a result, we can compute the variance of the unbiased CFO estimator, $\hat {\mu }$, as

60 $$\begin{array}{*{20}l} \text{Var}(\hat{\mu}) \geq [(\mathcal{J})^{-1}]_{1,1}=\textrm{CRB}_{\mu}. \end{array} $$

Similarly, the variance of the unbiased STO estimator, $\hat {\tau }$, is derived as

61 $$\begin{array}{*{20}l} \text{Var}(\hat{\tau}) \geq [(\mathcal{J})^{-1}]_{2,2}=\textrm{CRB}_{\tau}. \end{array} $$

Finally, for the *l*th tap of the channel, the lower bound of the unbiased estimator can be obtained as

62 $$\begin{array}{*{20}l} \text{Var}(\hat{h}[l])=& \text{Var}(\hat{h}_{R}[l])+\text{Var}(\hat{h}_{I}[l]) \geq [(\mathcal{J})^{-1}]_{l+2,l+2}\\ =&+[(\mathcal{J})^{-1}]_{Q+l+2,Q+l+2}\textrm{CRB}_{h[l]}. \end{array} $$

The lower bound on the average variance of the CIR estimator over different taps can be obtained by assuming that the tap estimates are independent. Then, we can write

63 $$\begin{array}{*{20}l} \textrm{CRB}_{\mathbf{h}} & \,=\, \frac{1}{Q}\left(\text{tr}[\!(\mathcal{J})^{-1}(\boldsymbol{\theta})]\!-[\!(\mathcal{J})^{-1}(\boldsymbol{\theta})]_{1,1}\!- [\!(\mathcal{J})^{-1}(\boldsymbol{\theta})]_{2,2}\right). \end{array} $$

It is worth emphasizing that in general the entries of the vectors ***κ***, ***ε***, ***ϕ***, ***ρ*** and ***χ*** are not negligible, i.e., there is a coupling between the estimation errors that can be achieved for *μ*, *τ*, and **h**. This means that, for example, the CRB on *μ* with no channel knowledge will be greater than the one obtained with a known channel, which could be directly computed as the inverse of the first entry of the FIM, i.e. (*J*_1,1_)^−1^. This also applies to the STO and CIR estimators with or without knowledge of other parameters. Furthermore, as it has been mentioned in [38], the derivations imply that the CRB is a function of the particular channel realization. This has also been observed through simulations. Also, it should be noted that in the derivation and implementation of the CRB, the simplifications of Section 3.3 are not introduced, i.e., on a given output symbol, the impact of all the subbands are taken into account.

## Performance evaluation

This section begins with the simulation setup and parameter settings for performance evaluation of the proposed method compared to the existing one, followed by presentation and discussion on their estimation results and complexity evaluation.

### Methodology

The prototype filter of the transceiver system is obtained using the frequency sampling technique with overlap factor *K*=4 as described in [31] and used in [29]. The data are modulated to a 4-QAM constellation. The input sampling frequency is *F*_*s*_=175 kHz corresponding to a channel bandwidth of *MF*_*s*_=11.2 MHz.

To obtain BER that are more representative of a practical digital communications system, a punctured convolutional channel coding scheme is applied to the information sequence with the overall rate of 2/3 by using constraint lengths vector [ 5 4] and vector of function generators [ 23 35 0; 0 5 13]. A frequency selective wireless channel is used with *Q*=8 randomly generated coefficients *h*[*l*] based on the ITU-Vehicular A channel guidelines [39]. The channel is assumed constant during the transmission of a burst but changes over different transmissions^9^,^10^. During each transmission, the STO and CFO obey a uniform distribution within the intervals $\left [-\frac {T_{s}}{4} \; \frac {T_{s}}{4}\right ]$ and $\left [-\frac {F_{s}}{4} \; \frac {F_{s}}{4}\right ]$. The root mean squared error (RMSE) and BER results are obtained by running 500 independent Monte-Carlo simulations for given values of the SNR per bit. The latter is expressed as *E*_*b*_/*N*_0_, where *E*_*b*_ denotes the bit energy and *N*_0_ is the noise power spectral density level. Regarding the implementation results of [29], we follow the estimation and equalization algorithms and structure precisely as described in the paper. This method is referred to as “Stitz” in Figs. 4, 5, 6 and 7.

**Fig. 3:**
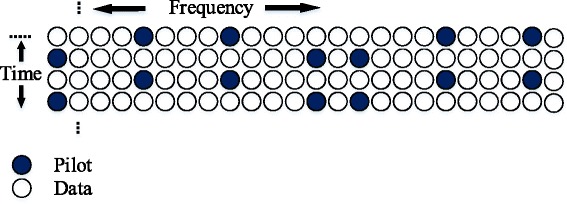
Pilot distribution in WiMAX, DL-PUSC configuration [29]

We compare the proposed method to [29], one of a few works that estimate all the three aforementioned parameters in their paper. To this end, two different distributions of pilots are considered in the implementation of the proposed method. In the first distribution, the pilots are scattered within each burst as mentioned in [29]. Specifically, the size of the transmitted bursts in time and frequency are according to DL-PUSC configuration as illustrated in Fig. 3, with *M*=64 subcarriers and *N*=54 input symbols. The other distribution, which also uses *M*=64 subcarriers, adopts a full preamble of pilot tones of length *T*=4, followed by *N*−*T*=50 data symbols. In this way, the two bursts share the same data rate and hence are comparable. The consideration of these two different distributions of pilot tones is useful to assess the performance of different transmission modes.

### Results and discussion

The performance and the complexity comparison of the proposed estimator vis-a-vis the benchmark are, respectively, presented in this subsection.

#### Estimation results

**Fig. 4:**
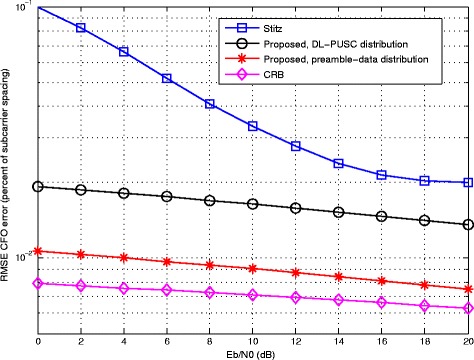
Comparative RMSE error of CFO estimation versus input *E*_*b*_/*N*_0_ with STO = 2.5% and Rayleigh channel

Figure 4 compares the proposed method with [29] in terms of RMSE of CFO as a function of *E*_*b*_/*N*_0_. The average CRB of the CFO estimation is also presented in the figure. The estimation is jointly performed in the presence of other sources of error, namely, a fixed STO of 2.5*%* with respect to *T*_*s*_, and Rayleigh fading channel as described earlier. The figure indicates that the proposed method not only outperforms the other method as implemented with a preamble of full 64×4 pilot tones, but also, is capable of significant improvement when adopting the burst structure of DL-PUSC. Especially in the former case, the performance of the proposed estimator is very close to the average CRB as a lower bound.

**Fig. 5:**
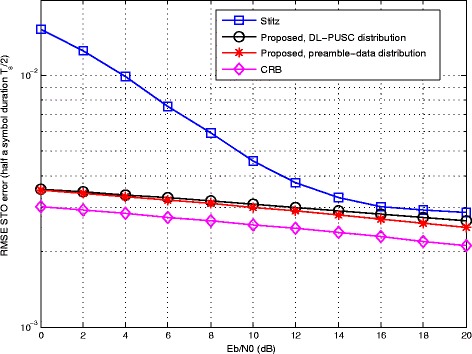
Comparative RMSE error of STO estimation versus input *E*_*b*_/*N*_0_ with CFO = 5% and Rayleigh channel

Comparison of the two methods and the average CRB in terms of RMSE of STO is depicted in Fig. 5, where a fixed CFO of 5% and the multi-path channel are used. The superior performance of the proposed method implemented in both configurations can be seen in the figure. Again, the best result belongs to the one based on a full grid of pilot tones followed by the data; although, the proposed method remarkably reduces the estimation error with the same burst structure as in [29]. Similar to the previous figure, the average CRB runs closest to the full-preamble case.

**Fig. 6:**
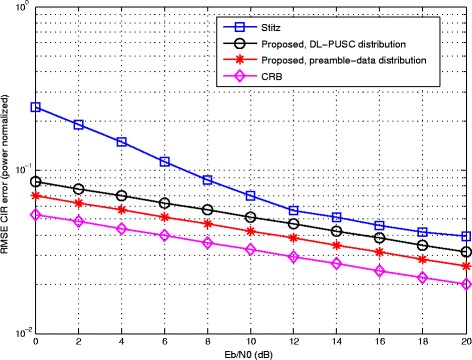
Comparative RMSE error of channel estimation versus input *E*_*b*_/*N*_0_ with CFO = 5% and STO = 2.5%

In Fig. 6, the RMSE of CIR, in the presence of fixed CFO and STO, is depicted for the three implementations along with the average CRB. Similar to the previous figures, the proposed method achieves a lower estimation error in both configurations. The figure indicates that the CIR estimator performs very close to the average CRB over different channels.

It is worth mentioning that, in this work, all the CRB terms are inversely proportional to $\sigma ^{2}_{v}=\sigma ^{2}_{\zeta ^{d}}+\frac {\sigma ^{2}_{\eta }}{2}$. This explains the mild slope of the estimation error graphs with respect to *E*_*b*_/*N*_0_. In other words, in our range of interest of *E*_*b*_/*N*_0_, the dominant term is the power of the data interference, $\sigma ^{2}_{\zeta ^{d}}$ rather than the noise power. However, our experiments show that the noise power effect begins to rise when applying lower SNR values with an abrupt increase in the estimation error around *E*_*b*_/*N*_0_=−10 *dB*.

**Fig. 7:**
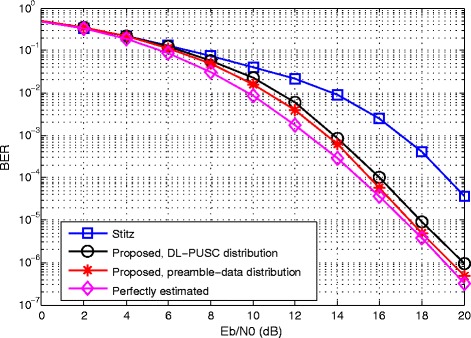
Comparative coded BER versus input *E*_*b*_/*N*_0_ in the presence of CFO, STO and channel estimation

The coded BER performance of the methods, after estimation and compensation, are compared in Fig. 7. The figure indicates that, in both configurations, the proposed method is capable of a significant decrease in BER of the system especially at high input *E*_*b*_/*N*_0_.

#### Complexity evaluation and discussion

The complexity of the two methods is compared in Table 2 in terms of the running time needed for the processing of a burst transmission of size 64×54. For the proposed method, three different implementations are considered as follows:

**Table 2:** Comparative running time and RMSE estimation errors of the simplified versions of the proposed method vs. Stitz for *E*_*b*_/*N*_0_=20 *dB* [29]

	Prop 0	Prop 1	Prop 2	Stitz
Running time (s)	4617	65	28	15
RMSE CFO	1.1×10^−2^	1.2×10^−2^	1.4×10^−2^	2.0×10^−2^
RMSE STO	2.2×10^−3^	2.4×10^−3^	2.7×10^−3^	2.8×10^−3^
RMSE CIR	2.9×10^−2^	3.1×10^−2^	3.4×10^−2^	4.0×10^−2^

- *Prop 0*: the proposed method with the basic simplification in (35)-(36);
- *Prop 1*: as above with additional simplification (37);
- *Prop 2*: as Prop 1, but by considering only the first three channel taps in computing the pilot contribution in (27).

In addition, Table 2 includes the RMSE figures of CFO, STO and CIR (for *Eb*/*N*0=20 *dB*), to illustrate the achievable trade-off between estimation accuracy and computational complexity. The RMSE figures are obtained based on the average of 500 burst transmissions of size 64×54 with the scattered pilot distribution as described in Section 5.1, on an ordinary quad-core PC running MATLAB 2016 b. The table indicates that the performance degradation due to the suggested simplifications is rather small, i.e., on the order of 10% for Prop 1 and 25% for Prop 2. Furthermore, by employing all the foregoing simplifications, the obtained running time of the proposed method comes within about two times that of the benchmark, which implies that, by using state-of-the-art DSP technology, the running time of the proposed method remains practical. It should be noted that, as can be seen from the results in Subsection 5.2.1, in mid and specially low SNR regime, the performance gap between the two methods is much larger than that presented in Table 2 for *E*_*b*_/*N*_0_=20 *dB*. Hence, considering the performance gain of the proposed estimator presented in Section 5.2, the complexity compromise seems justifiable.

From a theoretical perspective, a key advantage of the proposed ML-based approach is to offer a unified treatment leading to a compact solution format (i.e., near closed form) for the joint estimation of CFO, STO and CIR in OFDM/OQAM systems, in contrast to making use of different signal processing techniques (correlation, phase estimation, etc.) for the treatment of different impairment sources. Another important motivation behind the ML-based approach lies in its asymptotic optimality, as observed in many practical situations of interest, under conditions of high SNR or long observation time [37].

From a practical perspective, the proposed joint ML-based estimator leads to significant improvements in estimation accuracy compared to existing methods, as demonstrated by the simulation results in this section. Specifically, over the complete range of SNR considered (from 0 to 20dB), the proposed estimator achieves the best performance for the three types of parameters, i.e., CFO, STO and CIR coefficients. For each parameter type, the resulting RMSE obtained with the joint-ML estimators comes within 1dB of the CRB. In turn, the improved estimation accuracy results in lower BER for the OFDM/OQAM transceiver with ML-based compensation.

The main limitation of the proposed method is the additional computational burden. Indeed, the calculation of the joint ML estimator involves several matrix operations and a two-dimensional search over the CFO and STO space. However, by allowing a number of possible simplifications to reduce the processing time as discussed in Section 3.3, the proposed method offers a trade-off between complexity and performance.

## Conclusions

A new general pilot-based ML joint estimation method for OFDM/OQAM systems has been developed and evaluated. The CFO, STO, and CIR effects have been jointly estimated and compensated. The CRB on the joint estimator variance was also derived and implemented as a reference to evaluate the performance of the proposed algorithm. The comparison has been made with a highly-cited method among the few research papers of the same focus. The results have shown the significant improvement that the proposed method offers in both transmission modes, i.e., as scattered pilots in data and as a full preamble of pilot tones followed by the data. As it was observed on the figures, the proposed estimator, especially when used in a full-preamble setup, performs close to the CRB and can robustly estimate the CFO, STO, and CIR. Furthermore, by comparing the performance and running times of the simplified versions of the proposed method to those of the benchmark, we conclude that the former provides a significant improvement over the estimation result while maintaining computational complexity in a feasible range. This, in turn, offers a useful trade-off between performance and complexity.

## Appendix A: Statistical properties of the subband noise *η*_*k,n*_

Let *η*[*m*] be a zero-mean complex circular AWGN process with variance $\sigma ^{2}_{\eta }$. By definition, we have,

64 $$\begin{array}{*{20}l} \mathrm{E} \: \{\eta[m]\eta[m']\}\,=\,0\:, \: \: \mathrm{E} \: \{\eta[m]\eta^{*}[m']\}\,=\,\delta_{mm'}\sigma^{2}_{\eta}, \: \: \forall m,m'. \end{array} $$

According to (15) and (7), we can write

65 $$\begin{array}{*{20}l} \eta_{k,n} = &\ \mathfrak{R} \left\{\theta^{*}_{k,n}\sum\limits_{m} \eta[m]f^{*}_{k}\left[m-\frac{nM}{2}\right] \right\} \end{array} $$

66 $$\begin{array}{*{20}l} = &\ \frac{1}{2} \left\{\theta^{*}_{k,n}\sum\limits_{m} \eta[m] f^{*}_{k}\left[m-\frac{nM}{2}\right]\right.\\& \left.+ \theta_{k,n}\sum_{m} \eta^{*}[m] f_{k}\left[m-\frac{nM}{2}\right] \right\}. \end{array} $$

Due to the linearity of this expression, it follows that *η*_*k,n*_ is a (real) Gaussian random variable with zero mean. For the second order moments we have,

67 $$\begin{array}{*{20}l} \mathrm{E} \: \{ \eta_{k,n}& \eta_{k',n'}\} \\=&\: {\textcircled{1}}+ {\textcircled{2}}+ {\textcircled{3}}+ {\textcircled{4}}, \end{array} $$

68 $$\begin{array}{*{20}l} {\textcircled{1}} =& \frac{1}{4}\theta^{*}_{k,n}\theta^{*}_{k',n'}\sum\limits_{m} \sum\limits_{m'} \underbrace{\mathrm{E}\{\eta[m]\eta[m']\}}_{0} f^{*}_{k}\left[m-\frac{nM}{2}\right] \\&f^{*}_{k'}\left[m'-\frac{n'M}{2}\right]=0, \end{array} $$

69 $$\begin{array}{*{20}l} {\textcircled{2}} =& \frac{1}{4}\theta^{*}_{k,n}\theta_{k',n'}\sum\limits_{m} \sum\limits_{m'} \underbrace{\mathrm{E}\{\eta[m]\eta^{*}[m']\}}_{\sigma^{2}_{\eta}\delta_{mm'}}f^{*}_{k} \left[m-\frac{nM}{2}\right] \\&f_{k'}\left[m'-\frac{n'M}{2}\right] \end{array} $$

70 $$\begin{array}{*{20}l} =& \frac{1}{4}\sigma^{2}_{\eta}\theta^{*}_{k,n}\theta_{k',n'} \sum\limits_{m} f^{*}_{k} \left[m-\frac{nM}{2}\right]f_{k'}\left[m-\frac{n'M}{2}\right], \end{array} $$

71 $$\begin{array}{*{20}l} {\textcircled{3}}=& \text{complex conjugate of} \: {\textcircled{2}}, \end{array} $$

72 $$\begin{array}{*{20}l} {\textcircled{4}}=& \text{complex conjugate of} \: {\textcircled{1}}. \end{array} $$

Therefore,

73 $$\begin{array}{*{20}l} \mathrm{E} \: \{ \eta_{k,n} \eta_{k',n'}\} =&\frac{\sigma^{2}_{\eta}}{4}\theta^{*}_{k,n}\theta_{k',n'} \sum\limits_{m} f^{*}_{k}\left[m-\frac{nM}{2}\right] \\&f_{k'}\left[m-\frac{n'M}{2}\right]+ {\textcircled{3}} \end{array} $$

74 $$\begin{array}{*{20}l} =& \frac{\sigma^{2}_{\eta}}{2}\mathfrak{R}\left\{\theta_{k,n}\theta^{*}_{k',n'} \sum\limits_{m} f_{k}\left[m-\frac{nM}{2}\right]\right. \\&\left.f^{*}_{k'}\left[m-\frac{n'M}{2}\right]\right\}=\frac{\sigma^{2}_{\eta}}{2}\delta_{kk'}\delta_{nn'}. \end{array} $$

where we have used the orthonormality relation (10) for OFDM/OQAM systems.

## Appendix B: Statistical properties of the data interference term $\zeta _{k,n}^{\;d}$

**Fig. 8:**
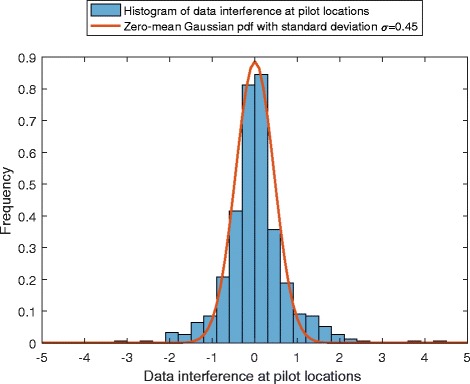
Histogram of $\zeta _{k,n}^{d}$ at pilot locations in presence of fading channel, STO and CFO

To further investigate the Gaussian assumption of the data interference, the histogram of data contribution terms at pilot locations is obtained and depicted in Fig. 8. To acquire pure data interference, a data burst of the same structure and size as described in Section 5.1 is transmitted and received where pilots are replaced by zero tones. No noise was added to the system. The histogram of data interference terms at all the 512 pilot locations is illustrated. A Gaussian distribution with zero mean and a standard deviation of *σ*=0.45 is also depicted for comparison. The figure suggests that the statistical distribution of the data interference terms tends towards the PDF of a Gaussian distribution.

In addition, to examine the covariance of these data interference terms at pilot locations, let the data symbols $d_{k,n}^{\;d}$ be i.i.d. (real) random variables with zero mean and variance $\frac {1}{2}\sigma ^{2}_{x}$. By definition, we have

75 $$\begin{array}{*{20}l} \mathrm{E}\left\{ d^{\;d}_{k',n'} d^{\;d}_{\bar{k}',\bar{n}'}\right\} & =\frac{1}{2}\delta_{k'\bar{k}'}\delta_{n'\bar{n}'}\sigma^{2}_{x}. \end{array} $$

According to (22) we can write

$$\begin{array}{*{20}l} \zeta_{k,n}^{\;d}&=\sum\limits_{(k',n')\notin \mathcal{P}} d_{k',n'}^{\;d} \mathfrak{R}\left\{\sum\limits_{l=0}^{Q-1}h[l] \gamma^{k',n'}_{k,n}(l,\mu_{0},\tau_{0}) \right\}.  \end{array} $$

Following the observation above in the opening of this appendix, by invoking the central limit theorem and the assumptions made on $d_{k,n}^{\;d}$, it follows from (75) that $\zeta _{k,n}^{\;d}$ can be modeled as a zero-mean (real-valued) Gaussian random process. For the second order moments, it follows from (75) that

76 $$\begin{array}{*{20}l} \mathrm{E} \: \left\{\zeta_{k,n}^{\;d} \zeta_{\bar{k},\bar{n}}^{\;d} \right\} =&\ \frac{\sigma_{x}^{2}}{2} \sum\limits_{(k',n') \notin \mathcal{P}} \mathfrak{R} \left\{ \sum_{l=0}^{Q-1} h[l]\gamma^{k',n'}_{k,n}(l,\mu_{0},\tau_{0})\right\} \\ &\mathfrak{R} \left\{ \sum\limits_{\bar{l}=0}^{Q-1} h[\bar{l}]\gamma^{k',n'}_{\bar{k},\bar{n}}(\bar{l},\mu_{0},\tau_{0})\right\}. \end{array} $$

We note that the filter-bank response term $\gamma ^{k',n'}_{k,n} (l,\mu _{0},\tau _{0})$ tend to be non-zero only in the vicinity of (*k,n*). Therefore, for a given (*k*^′^,*n*^′^) either $\gamma ^{k',n'}_{k,n}(l,\mu _{0},\tau _{0})$ or $\gamma ^{k',n'}_{\bar {k},\bar {n}}(l,\mu _{0},\tau _{0})$ tend to be zero. Hence,

77 $$\begin{array}{*{20}l} \text{If}\: (\bar{k},\bar{n})&\neq ({k},{n}) \Longrightarrow \mathrm{E} \: \left\{ \zeta_{k,n}^{\;d} \zeta_{\bar{k},\bar{n}}^{\;d} \right\} \approx 0, \end{array} $$

78 $$\begin{array}{*{20}l} \text{If}\: (\bar{k},\bar{n})&= ({k},{n}) \Longrightarrow \mathrm{E} \: \left\{ \zeta_{k,n}^{\;d} \zeta_{\bar{k},\bar{n}}^{\;d} \right\} =E\left\{(\zeta^{d}_{k,n})^{2}\right\}, \end{array} $$

for which we can write

79 $$\begin{array}{*{20}l} E\left\{\!\left(\zeta^{d}_{k,n}\right)^{2}\right\}\!=\frac{\sigma_{x}^{2}}{2}\! \sum\limits_{(k',n') \notin \mathcal{P}}\!\left(\!\mathfrak{R}\!\left\{\!\sum\limits_{l=0}^{Q-1}\!h[l]\gamma^{k',n'}_{k,n}(l,\mu_{0},\tau_{0})\!\right\}\!\right)^{2}. \end{array} $$

**Fig. 9:**
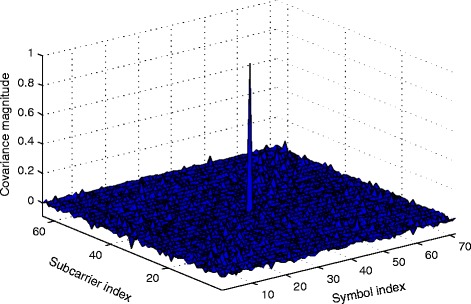
Covariance of data interference at a pilot location with data interference in other pilot locations

It can be seen from (79) that the variance of the data interference term depends on the particular channel realization and the unknown CFO and STO parameters. However, since the filter bank response $\gamma ^{k',n'}_{k,n}(l,\mu _{0},\tau _{0})$ does not profoundly vary with the aforementioned parameters, the effect of the channel has been approximated by a fixed gain. This approximation can be further inspected by the simulation result in Fig. 9. The figure illustrates the covariance of data interference contributions with respect to one another in the simulated OFDM/OQAM system with the scattered pilot-data distribution considered in this work. More specifically, it depicts the covariance of a data interference contribution at a pilot location with data interference contributions to other pilot locations in presence of 8-tap Rayleigh fading channel, 20% CFO, 25% STO with no noise. For the considered pilot-data distribution with length of 160 OQAM symbols, the second largest covariance is less than 10% of the peak value. Although only one sample is presented here, we were able to verify the consistency of this result for different values of CFO and STO with various fading channels, indicating that the observed maximum off-center covariance value does not exceed 14% of the peak value. This result is consistent with our approximation above in (77) and (79).

## Footnotes

1 The authors’ intention in the performance comparison is not to include the existing estimators in which only one or two of the aforementioned error sources are considered.

2 For convenience in our analysis, *G*_*k*_(*z*) is assumed non-causal; although, in practice, causality can be restored simply by introducing an appropriate delay in the receiver.

3 The precise orthogonality condition corresponding to the specific configuration of OFDM/OQAM system adopted in this work does not exist in the literature. However, by applying appropriate modifications pertained to the changes in the configuration, the presented orthogonality condition can be derived.

4 In practice, non-integer STO can be modeled with the help of an ideal low-pass interpolation filter (see Section 3.4).

5 Due to the inherent property of OFDM/OQAM, as implied in (10), which only sustains real orthogonality, estimation is performed on the received pilots after extracting the real part.

6 In this work, we develop a single-tap-per-subcarrier equalizer, although generalizations to other, more advanced types of equalizers are possible. This simple equalization scheme inverts the channel at the center frequency of the corresponding subcarrier and it works well in mildly selective channels as long as the number of subcarriers is sufficiently large [36].

7 This is only valid for the PHYDYAS filter. There are other prototype filter functions in discussion for OFDM/OQAM systems which may require a different value for *β* [34].

8 It is worth emphasizing that the CRB derived in this section is based on the approximation of Gaussian distribution for the data interference term $\zeta ^{\;d}_{k,n}$; hence, it provides a benchmark for the performance of the estimators developed on such an assumption. Nevertheless, as it can be seen later, the proposed estimator obtains satisfactory results by a significant improvement over the existing method.

9 The authors have also evaluated the performance of the proposed and the existing method by adopting a time-varying fading channel in contrast to the static channel used in the following simulations. However, the results show the same relative trend among the curves corresponding to the two methods and the two pilot distribution schemes described in this section.

10 Although, in the proposed method, the channel length is supposed to be known, the authors have investigated a mismatch in the presumed channel length with the actual one and did not find a significant performance degradation in CFO and STO estimation. Nevertheless, the effect of such a mismatch on channel estimation and BER performance is considerable. In a practical system, to mitigate this problem, as a conventional way to make the channel estimation task more robust, the channel length can be matched to a worst-case scenario and may be modified adaptively [40].
